# Supplementary material for: Creation of an ustekinumab external control arm for Crohn’s disease using electronic health records data: A pilot study
Source: PLoS One. 2023 Mar 2;18(3):e0282267. doi: 10.1371/journal.pone.0282267 (PMC9980824; doi:10.1371/journal.pone.0282267)
Supplement: S1 File — (DOCX) [file pone.0282267.s002.docx]

**Supporting Information**

Table of Contents:

S1. Definitions of informatically-ascertained CDAI elements

S2. Calculation of the CDAI

S3. Abstraction definitions

**S1. Definitions of informatically-ascertained CDAI elements**

For each of the below extraintestinal manifestation variables, the diagnosis was considered present (and corresponding points were assigned) if the below codes were found within time windows corresponding to baseline, week 12, and week 24.

*Arthritis/arthralgias*:

- ICD-9-CM: 710*-719*
- ICD-10-CM: M04*-M14.*

*Iritis/uveitis:*

- ICD-9-CM: 364*
- ICD-10-CM: H20*

*Erythema nodosum, pyoderma gangrenosum, aphthous stomatitis:*

- ICD-9-CM: 686.01, 695.2, 528.2
- ICD-10-CM: L88*, L52*, K12.0

*Anal fissure, perianal fistula, perianal abscess*:

- ICD-9-CM: 565*/566*
- ICD-10-CM: K60*/K61*

*Other fistula*:

- ICD-9-CM 569.81
- ICD-10-CM K63.2

Abdominal mass was assumed absent for all patients. Other variables were obtained from structured data as follows:

*Weight, fever/temperature > 100^o^ F:* Ascertained by query of patient vitals tables

*Hematocrit*: Ascertained by query of patient labs tables

*Antidiarrheals or Opiates*: Ascertained by query of patient medications tables corresponding to the following medications: diphenoxylate-atropine (Lomotil), loperamide (Imodium), Oxycodone, Oxycontin, Percocet, Norco, Vicodin, Hydrocodone

For each of the diagnoses above, the absence of any corresponding diagnosis code was treated as positive evidence of absence (i.e. no missing data in these variables by definition). Similarly, the absence of a medication listing for an antidiarrheal or opiate was treated as positive evidence of absence.

*Ideal body weight*: Patient heights were ascertained by query of the patient vitals tables. This height and gender were used to determine the ideal body weight according to the following table provided by the study sponsor.

| Actual Height (cm) | Men | Women |
| --- | --- | --- |
| 137.2 to 138.3 | 54.4 | 48.5 |
| 138.4 to 139.6 | 54.9 | 49 |
| 139.7 to 140.9 | 55.3 | 49.4 |
| 141.0 to 142.1 | 55.8 | 49.9 |
| 142.2 to 143.4 | 56.2 | 50.3 |
| 143.5 to 144.7 | 56.7 | 50.8 |
| 144.8 to 146.0 | 57.1 | 51.2 |
| 146.1 to 147.2 | 57.6 | 51.7 |
| 147.3 to 148.5 | 58 | 52.2 |
| 148.6 to 149.8 | 58.5 | 52.6 |
| 149.9 to 151.0 | 59 | 53.1 |
| 151.1 to 152.3 | 59.4 | 53.6 |
| 152.4 to 153.6 | 59.9 | 54.2 |
| 153.7 to 154.8 | 60.3 | 54.8 |
| 154.9 to 156.1 | 60.8 | 55.3 |
| 156.2 to 157.4 | 61.2 | 56 |
| 157.5 to 158.7 | 61.7 | 56.7 |
| 158.8 to 159.9 | 62.1 | 57.4 |
| 160.0 to 161.2 | 62.6 | 58 |
| 161.3 to 162.5 | 63 | 58.7 |
| 162.6 to 163.7 | 63.5 | 59.4 |
| 163.8 to 165.0 | 64.1 | 60.1 |
| 165.1 to 166.3 | 64.6 | 60.8 |
| 166.4 to 167.5 | 65.2 | 61.4 |
| 167.6 to 168.8 | 65.8 | 62.1 |
| 168.9 to 170.1 | 66.4 | 62.8 |
| 170.2 to 171.4 | 67.1 | 63.5 |
| 171.5 to 172.6 | 67.8 | 64.2 |
| 172.7 to 173.9 | 68.5 | 64.9 |
| 174.0 to 175.2 | 69.2 | 65.5 |
| 175.3 to 176.4 | 69.8 | 66.2 |
| 176.5 to 177.7 | 70.5 | 66.9 |
| 177.8 to 179.0 | 71.2 | 67.6 |
| 179.1 to 180.2 | 71.9 | 68.3 |
| 180.3 to 181.5 | 72.6 | 68.9 |
| 181.6 to 182.8 | 73.4 | 69.9 |
| 182.9 to 184.1 | 74.1 | 70.3 |
| 184.2 to 185.3 | 75 | 71 |
| 185.4 to 186.6 | 75.7 | 71.7 |
| 186.7 to 187.9 | 76.6 | 72.3 |
| 188.0 to 189.1 | 77.5 | 73 |
| 189.2 to 190.4 | 78.4 | 73.7 |
| 190.5 to 191.7 | 79.1 | 74.4 |
| 191.8 to 192.9 | 80.2 | 75.1 |
| 193.0 to 194.2 | 81.2 | 75.7 |
| 194.3 to 195.5 | 82.1 | 76.4 |
| 195.6 to 196.8 | 83 | 77.1 |
| 196.9 to 198.0 | 83.9 | 77.8 |
| 198.1 to 199.3 | 84.8 | 78.5 |
| 199.4 to 200.6 | 85.7 | 79.1 |
| 200.7 to 201.8 | 86.6 | 79.8 |
| 201.9 to 203.1 | 87.5 | Not applicable |
| 203.2 to 204.4 | 88.5 | Not applicable |
| 204.5 to 205.6 | 89.4 | Not applicable |
| 205.7 to 206.9 | 90.3 | Not applicable |
| 207.0 to 208.2 | 91.2 | Not applicable |
| 208.3 to 209.5 | 92.1 | Not applicable |
| 209.6 to 210.7 | 93 | Not applicable |
| 210.8 | 93.9 | Not applicable |

**S2. Calculation of the CDAI**

Calculation generally followed the formula as originally published by Best et al (1976) with the following modifications to better match the statistical analysis plan of TRIDENT:

- Weight loss was calculated as a percentage relative to the ideal body weight. Weight loss calculated to be less than -10% were truncated to -10%
- 30 points were assigned if the patient had any evidence of antidiarrheal therapy or opiate use within a given time window

**S3. Abstraction Definitions**

The below protocol was used to support Phases 2-6 as described in the methods section. Prior to its use, it was distributed in advance to the study sponsor and to all annotators for comment and revision.

**General Comments**

This chart review process requires that the reviewer understand the principles of clinical medicine and gastroenterology, the medical management of Inflammatory Bowel Disease (IBD), and various practical aspects of healthcare delivery. This knowledge is needed to properly interpret data found in the electronic health records (EHR). Although this expertise is typically found among gastroenterology clinicians with exposure to IBD patient management, it may also be developed in individuals with a less technical background following an initial period of close supervision.

In the review process, annotators should make use of the complete information available within the EHR. This includes notes, encounter-level data, scanned documents, endoscopy reports, global search, and patient-authorized clinical data shared from other sites where healthcare has been provisioned (i.e. Health Information Exchange data via *Care Everywhere* or equivalent).

Annotators are encouraged to take a ‘360 degree’ approach that relies on several independent sources of information rather than relying on a single source of truth. They are encouraged to anticipate the possibility of mis-annotation from ‘copy forwarding’, and to recognize sections of the clinical note more likely to be meaningfully annotated (e.g. the assessment/plan or impression section, or a de novo, free-texted nursing note) than others. They are encouraged to be conservative in the face of uncertainty. This may involve documenting certain fields as missing rather than available.

Annotators are recommended to use heuristics that incorporate existing knowledge about clinical medicine, common practice of medication usage in real-world practice settings, and healthcare delivery. These heuristics include understanding common frequencies of dosing, locations of where medication are delivered and whom might document them, as well as common and uncommonly prescribed combinations of IBD treatments among others. The use of this process makes the record review process somewhat more ‘organic’ and less ‘algorithmic’ or ‘deterministic’, but in our view substantially increases the relevant information content available within the EHR without sacrificing robustness or reproducibility.

This document does not aspire to be exhaustive and cover every edge case; we view this task as being essentially impossible and/or one that may bias the annotation process in unanticipated and undesirable ways. Rather, we anticipate that a combination of this guidance with individuals of sufficient training will lead to the consistent identification and annotation of medical records corresponding to Ustekinumab-treated Crohn’s Disease patients.

**Part 1 Annotations**:

Variables should be annotated in order; if a patient already merits exclusion from the target study based on an earlier variable, subsequent variables may be annotated NA at the discretion of the reviewer.

**MRN** (Integer):
Medical Record Number of the patient obtained by structured database query

**Annotated by** (character):
Initials of the chart annotator

**Confirmed dx** (Y, N, NA):
Annotate as Y if the patient was confirmed to have a diagnosis of Crohn’s disease at the time of Ustekinumab initiation, N if there is positive evidence for a different diagnosis (including IBD-unclassified), and NA if insufficient data.

The sources of this diagnosis should preferentially come from the gastroenterologist who either recommended or prescribed the drug, typically documented in a note from the clinic encounter associated with the prescription. Provisional diagnoses can also be taken from endoscopy reports done near the time of treatment initiation. Of note, this field corresponds to the diagnosis assigned by the clinician at the time of drug initiation and should not incorporate any subsequent diagnostic revision.

**Received UST** (Y, N, NA):
Annotate as Y if the patient was confirmed to have received Ustekinumab, N if not, and NA if there is insufficient information to confirm or deny receipt. In general the absence of information to support Ustekinumab initiation should be annotated as an N (i.e. use NA sparingly).

Confirmation of Ustekinumab receipt should typically be accompanied by the date of initiation (see next field). The gold-standard for receipt is an infusion center note (typically authored by a nurse) that provides a date and the details of this infusion encounter. It is sometimes accompanied by a timestamp in the form of a ‘medication administration record’ entry that is linked to these notes within the electronic health record.

An acceptable alternative is multiple sources of clinical documentation, typically by GI providers, that indicate a month and year of initiation.

**Date of UST** (YYYY-MM-DD, NA):
Provide the date that Ustekinumab was initiated. If a month and year are available but not the day, the day may be imputed to the 15^th^ day of that month (so that it is correct on average). If the month is unavailable (e.g. only the year), annotate this as NA.

As above, preferentially use infusion encounter or pharmacy notes to determine this. When unavailable can use documentation from the primary GI’s office, especially from clinic administration staff (who facilitate payor approval and schedule infusion appointments). Notes from non-GI clinicians (e.g. primary care, rheumatology, dermatology) can increase confidence in making this annotation. Rarely the reviewer may have to use the knowledge that this drug is typically dosed every 8 weeks in order to back calculate the precise day of infusion within a given month or week.

**Exact Date** (Y, N, NA): Annotate this as Y if the prior field corresponds to a precise date, or N if the day was imputed to ‘15’ due to inadequate documentation within the EHR. NA if the patient has been excluded by prior variables (i.e. patient does not have Crohn’s).

**Age >= 18** (Y, N, NA): Annotate a Y if the patient was aged 18 or older *at the time they received Ustekinumab*. Annotate as N otherwise, or NA if unable to determine (rare) or patient excluded by prior variables.

**NoRecentOrMajorSurgery** (Y, N, NA):

Annotate N if the patient had any small or large bowel resection within the 6 months prior to Ustekinumab infusion, or alternatively if they have had a history of >50% resection of the colon, or a clinical diagnosis of ‘short bowel syndrome.’ Annotate Y otherwise. If the patient record is deemed to have inadequate documentation related to these surgical events, annotate as NA.

**History of Biologic Intolerance/Refractoriness** (Y, N, NA):
Annotate as a Y if the patient has previously experienced
1) one of the following biologics (Infliximab, Adalimumab, Certolizumab, or Vedolizumab; Natalizumab, Golimumab, and other biologics are not listed in the TRIDENT trial protocol Attachment 1), and
2) either was not thought to have responded or lost response from the standpoint of Crohn’s disease (does not count if the patient received it for a different indication and Crohn’s disease activity were not explicitly evaluated or requiring of medical treatment).

Annotate as Y if the patient received one of the above biologics and experienced any intolerance to the medication that was thought to be related to the medication by any documenting clinician.

Document as N otherwise, or document as NA if insufficient data is available to make this determination.

**No TNFi<8wks** (Y, N, NA):
Annotate as N if the patient received a dose of a tumor necrosis factor inhibitor (TNFi) – for example Infliximab, Adalimumab, Certolizumab, or Golimumab – within 56 days of the date of Ustekinumab induction. Annotate Y if the patient has no history of prior TNFi use or if the last date of use was over 56 days prior to Ustekinumab induction.

Annotate as NA if the information within the EHR is inadequate to annotate as Y or N. At the reviewers discretion such patients may alternatively be imputed to N (so as to exclude them from any further consideration).

**No IV Steroids<3wks** (Y, N, NA):
Annotate as N if the patient received a dose of an IV corticosteroid, typically methylprednisolone or (less commonly) hydrocortisone within 21 days of Ustekinumab induction.

The annotator is reminded that these medications are almost exclusively administered in an inpatient/hospitalized setting. If the patient had a significant number of available primary care or gastroenterology clinician encounters within the 3 week window preceding or following Ustekinumab induction, this variable may be annotated as a Y. The logic underlying this is that the patient would be expected to have had documentation of such an event by a treating physician, and as such the absence of this documentation is treated as positive evidence of no hospitalization.

If the numbers of clinical encounters during this period are insufficient to confidently ascertain that the patient was not hospitalized, annotate this field as NA.

**No Vedo<16wks** (Y, N, NA):
Annotate as N if the patient received Vedolizumab within 112 days of the date of Ustekinumab initiation.

The annotator is reminded at as of the date of annotation for the present project (6/1/2020), Vedolizumab is only FDA approved as an infused drug and is not commercially available in a subcutaneous formulation – thus it is typically delivered in an infusion center and commonly accompanied by an note from an infusion nurse. He or she is also reminded that the drug is typically administered in 4 or 8 week intervals, and that the dosing frequency can typically be ascertained from clinical notes and/or documentation related to payor approval of dosing frequency.

Heuristics as suggested in the preamble of this document may be used to calculate the last date of Vedolizumab administration in order to annotate this variable.

If the patient has no history of Vedolizumab use, or received it outside of this window, annotate as Y. Annotate as NA if the EHR provides insufficient information to otherwise annotate this variable.

**Stable 5ASA 3wks** (Y, N, NA):
Annotate as Y if the patient has either not been on an oral 5-Aminosalicylate or derivative (e.g. mesalamine, olsalazine, balsalazide, sulfasalazine) in the 21 days prior to Ustekinumab, or has been on them at a stable dose for this period. Annotate as N if the patient has evidence of a fluctuating dose of this medication during the lead-in period. Annotate as NA if there is insufficient evidence of annotate otherwise.

Annotators are advised that this use of this medication is often poorly documented in clinical notes and may be captured properly during the medication reconciliation process. However, gastroenterologists (and occasionally rheumatologists) are the only clinicians who prescribe this medication and therefore the absence of specific mention in the clinical note or new medication order can be taken as evidence that the dose was stable (whether at a dose of 0mg or greater).

**Stable Abx 3wks** (Y, N, NA):
Annotate as Y if the patient has been on a stable dose of antibiotics as used for the primary treatment of Crohn’s Disease itself in the 21 days prior to Ustekinumab induction. If the patient has not been on antibiotics, annotate as Y. If the patient has been on antibiotics to treat an infection (infectious diarrhea, abscess, sepsis) this does not disqualify the patient. If the patient has been on antibiotics to treat a draining fistula, this would could as treatment of Crohn’s disease. Primary does not imply that it is the only treatment, but rather the purpose of that treatment. Annotate as N if the patient has received antibiotics to treat Crohn’s within the 21 days prior to Ustekinumab, or NA if insufficient data to determine this.

**Stable PO Steroids 3wks** (Y, N, NA):
Annotate as Y if the patient has either not been on an oral steroid (e.g. prednisone) in the 21 days prior to Ustekinumab, or has been on them at a stable dose under 40mg prednisone or 9mg budesonide (or equivalent) for this period.

If patients are on a higher dose than 40mg predisone equivalent, they should be annotated as a N. If they have been on either an increasing or decreasing dose during this period, they should be annotated as an N.

Annotators are advised that this medication is unevenly documented in the clinical record, and are advised to rely more strongly on active documentation of use or non-use as well as supporting documentation from prior GI visits as well as from other non-GI providers and/or providers outside of the host institution to obtain a more accurate picture.

In the absence of sufficient information, annotate this as NA.

**Stable MTX/6MP/AZA 12wks** (Y, N, NA):
Annotate as Y if the patient has either not been on an oral immunomodulator in the 84 days prior to Ustekinumab use, or N if so. If a patient has not been on immunomodulators previously and the GI who prescribes Ustekinumab does not indicate his or her intention to initiate this as co-treatment, then annotate this as N. If the patient has previously been on an immunomodulator and the notes are unclear as to whether or not the medication is being continued, annotate this as NA.

**NoOtherExclusion** (Y, N):

Annotate as Y if the patient should be excluded from the study for a reason not otherwise covered in the major criteria. Otherwise annotate as N. Blanks values and NAs will be assumed to represent an N.

**Likely Exclude** (Y, N, NA):
The purpose of this ‘flag’ is to potentially prioritize future, subsequent annotations in the event that this first pass results in too many cases.

Annotate this as Y if the patient has already been ‘excluded’ based on a major criterion from the variables covered thus far, or if he or she is likely to be excluded based on a variable not yet captured (but likely to be captured on the next round of review). Annotate as N if the chart reviewer has not identified any clear red flags suggesting exclusion. Annotate as NA for all other reasons.

**Borderline** (Y, N, NA):
The purpose of this flag is to prioritize future subsequent annotations in the event that this first pass results in too few cases.

Annotate as Y if the patient is currently listed as excluded but may be excluded for a relatively minor reason unlikely to substantially affect the outcome measurement (e.g. disease improvement on Ustekinumab at 12 or 24 weeks). These may be candidates for inclusion in a sensitivity analysis. Examples include fluctuating 5ASA dose (comparatively weak), exclusion based on recent drug exposure that was thought to be weakly effective or ineffective, etc. Annotate as N if the patient is clearly included or excluded, or NA otherwise.

**Part 1.1 annotations**:

**MRN** (Integer): Medical Record Number. This variable will be captured by an informatic pull

**Annotator** (character): Initials of the annotator

**Birthdate** (MM/DD/YYYY): Indicate the birthdate of the patient here

**Gender** (M, F, O, NA): Indicate the gender of the patient here, M for male, F for female, O for other, NA for missing data

**BioIR** (Y, N, NA):

Annotate as a Y if the patient has previously experienced
1) one of the following biologics (Infliximab, Adalimumab, Certolizumab, or Vedolizumab; Natalizumab, Golimumab, and other biologics are not listed in the TRIDENT trial protocol Attachment 1), and
2) either was not thought to have responded or lost response from the standpoint of Crohn’s disease (does not count if the patient received it for a different indication and Crohn’s disease activity were not explicitly evaluated or requiring of medical treatment).

Annotate as Y if the patient received one of the above biologics and experienced any intolerance to the medication that was thought to be related to the medication by any documenting clinician.

Document as N otherwise, or document as NA if insufficient data is available to make this determination.

**First Dose IV** (Y, N, NA):

Annotate Y if the patient received the first dose as an IV, N if the patient did not (i.e. subcutaneous), or NA if unable to determine.

If the patient is N or NA, no further annotations are necessary; the patient is to be excluded from the study.

**NoRecentOrMajorSurgery** (Y, N, NA):

Annotate N if the patient had any small or large bowel resection within the 6 months prior to Ustekinumab infusion, or alternatively if they have had a history of >50% resection of the colon, or a clinical diagnosis of ‘short bowel syndrome.’ Annotate Y otherwise. If the patient record is deemed to have inadequate documentation related to these surgical events, annotate as NA.

**Date of UST** (MM/DD/YYYY): Date the patient received the first dose of Ustekinumab. This can essentially be copied from your phase 1 annotations; but is only here to assist the annotator with coding other variables corresponding to an 8 week period lookback from this date.

**Bsln at UCSF** (Y, N, NA):

Annotate as Y if the patient was prescribed his or her first dose by a UCSF physician, N if not, or NA if unclear. Note that a prescription by a UCSF physician does not imply that the patient actually received the dose at a UCSF facility.

These patients typically are evaluated in an office visit or at endoscopy at the time of this prescription; as such this variable serves as a proxy for patients who are likely to have baseline and follow-up data available.

This criteria is not to be considered exclusionary at this time, so do continue to annotate other fields even if this is an N or NA. Rather, it serves as more of an indicator variable that will tell us how good the data capture is or isn’t for these patients. Based on this we will consider whether or not we will make this a hard eligibility criteria when we consider revising the first pass annotations to determine patient selection (e.g. include patients who did not meet the biologic washout period but did have good baseline data capture at UCSF).

**Bsln Daily Abd Pain** (Numeric, NA):

Within the -16 to 0 week period prior to the first dose of Ustekinumab, indicate patient’s level of daily abdominal pain according to the following scale:

0 for none, 1 for mild, 2 for moderate, 3 for severe.

If the notes indicate a range, report the mean (e.g. mild-moderate pain as a 1.5).

If otherwise not well documented, annotate as NA.

For situations in which there are multiple sources of truth within the 8 week period relevant to this data, prioritize reporting the one that is felt to be most reliable, followed by the data element closest to week 0.

**Date** **Bsln Daily Abd Pain** (MM/DD/YYYY):

Indicate the date of the note that the daily abdominal pain score was abstracted from. In the event that multiple dates contributed to a score, select the date closest to the date of infusion.

**Bsln Diarrhea** (Numeric, NA):

Within the -16 to 0 week period prior to the first dose of Ustekinumab, indicate the total number of bowel movements per day as reported at any point during the baseline period (-8 weeks to 0 weeks of the first Ustekinumab dose). If the notes report a range, indicate the average of the range. For instance, if the notes indicate 3-4 bowel movements daily, report 3.5 here.

Of note, this definition slightly defers from the trial protocol definition, which indicates the number of liquid or very soft stools daily. The rationale for this difference is that clinical notes tend to better capture total bowel movement frequency than they do characterize the quality of the bowel movement. Many patients with active Crohn’s disease tend to have loose bowel movements. Lastly this distinction is unlikely to impact downstream analyses, especially those that look for within-subject differences over time.

For situations in which there are multiple sources of truth within the 8 week period relevant to this data, prioritize reporting the one that is felt to be most reliable, followed by the data element closest to week 0.

If the notes do not explicitly indicate number of bowel movements during this period, but if notes from before and after this period indicate a stable frequency of bowel movements, then can use heuristics to report this during the baseline period.

If the patient has an ostomy, then annotate this as NA and flag this chart (these may ultimately be excluded). If the patient has an ileal pouch-anal anastomosis (also known as a ‘J-Pouch’, a surgery following complete removal of the rectum and colon), ok to report the number of bowel movements but also flag this chart. If otherwise not well documented, annotate as NA.

**Date** **Bsln Diarrhea** (MM/DD/YYYY):

Indicate the date of the note that the stool frequency was abstracted from. In the event that multiple dates contributed to a score, select the date closest to the date of infusion.

**Bsln WellBeing** (Numeric, NA):

Within the -16 to 0 week period prior to the first dose of Ustekinumab, score the patient’s apparent well-being on the following scale:

0 = generally well, 1 = slightly under par, 2 = poor, 3 = very poor, 4 = terrible

This variable is generally more difficult to annotate and may require some degree of annotator ‘reading between the lines’ in order to perform mapping to this scale. This variable is associated with the existing variables (abdominal pain, diarrhea) but also encompasses other inputs such as fatigue, subjective fevers, anxiety, loss of appetite, and other symptoms.

Patients who are symptom-free should be annotated as a 0. The presence of mild symptoms (occasional abdominal cramping, some loose stools) likely reflects a 1. Patients with significant symptoms, chronic use of opiates, steroids, seeing multiple specialists (mental health, nutrition/dieticians, rheumatology/dermatology) may be annotated as a 2. Patients who are hospitalized should be considered at least a 3, with patients who are suffering concomitant infection/sepsis and/or weight loss considered for a 4. For patients who appear to fit a range (e.g. between a 2 and 3), report the average (e.g. 2.5).

For situations in which there are multiple sources of truth within the 8 week period relevant to this data, prioritize reporting the one that is felt to be most reliable, followed by the data element closest to week 0.

**Date** **Bsln Wellbeing** (MM/DD/YYYY):

Indicate the date of the note that the wellbeing was abstracted from. In the event that multiple dates contributed to a score, select the date closest to the date of infusion.

**Bsln CRP** (Numeric, NA):

Within the -16 to 0 week period prior to the first dose of Ustekinumab, report the serum c-reactive protein level (in mg/L). Report the value closest to week 0. Note the units: this lab is sometimes reported as mg/dl, and under these scenarios has two decimal places rather than one.

Note the longer period of time for this baseline value; the rationale for this is that 1) this is a high-importance data element, 2) many patients end up having a prolonged period of prior treatment failure before receiving Ustekinumab so it might be more legitimate to carry forward this value, and 3) we can use the date field to refine this field by proximity to week 0 after the fact.

**Date Bsln CRP** (MM/DD/YYYY):

Indicate the date of the test (received, not ordered or resulted) corresponding to the crp reported in the prior field.

**Bsln Calprotectin** (Numeric, NA):

Within the -16 to 0 week period prior to the first dose of Ustekinumab, report the fecal calprotectin (in mcg/g). Report the value closest to week 0.

As with crp, we are using a longer window of time here in order to maximize the capture of this less commonly ordered (but highly informative) variable. If the variable proves to be too sparse we may decide to exclude it.

**Date Bsln Calprotectin** (MM/DD/YYYY):

Indicate the date of the test (received, not ordered or resulted) corresponding to the fecal calprotectin reported in the prior field.

**Bsln Steroid use** (Y, N, NA):

Indicate Y if the patient was taking steroids at the time of Ustekinumab induction, N if the patient was not, or NA if insufficient data to determine this. Use the Phase 1.1 document as appropriate to annotate this.

**Disease Location** (I, IC, C, O, NA):

Indicate if the patient has ileal disease (I), ileocolonic (IC), colonic (C), or other (O, e.g. isolated perianal). This designation is inherently ambiguous in part due to the lack of universal agreement regarding the interpretation of ileocecal valve involvement, lack of clarity regarding need for (and interpretation of) histological specimens as compared to gross endoscopic involvement, etc. The study sponsors have indicated that they intend to combine ileal and ileocolonic groups into a single category (to be distinguished from colonic).

As such, it is acceptable to simply use the designation indicated in the clinical notes at the time of Ustekinumab treatment without a need to refer to the original documentation (typical referral notes from other providers, with or without supporting endoscopy/pathology data). This is most likely to be consistent with how study clinicians are annotating this in the setting of prospective trials.

**Diagnosis** **Year** (YYYY, NA):

Indicate the year that the diagnosis was confirmed by a treating clinician. This is typically done by colonoscopy with biopsies or less commonly surgical explant histopathology. Many patients are diagnosed elsewhere and referred in so search for the earliest GI note and consider looking in the scanned clinical documents tab to identify outside docs sent in around the time of the referral to UCSF.

**NoOtherExclusion** (Y, N):

Annotate as N if the patient should be excluded from the study for a reason not otherwise covered in the major criteria. Otherwise annotate as Y. Blanks values and NAs will be assumed to represent an N.

**Likely Exclude** (Y, N):

Annotate as a Y if the patient fails eligibility criteria based on the reassessment done here (including recent or major bowel surgeries, or first dose not given IV).

**Part 2 Annotations**:

**W12AbdPain** (Numeric, NA):

Within the week 10-14 period with respect to the patient’s dose of IV Ustekinumab, indicate patient’s level of daily abdominal pain according to the following scale:

0 for none, 1 for mild, 2 for moderate, 3 for severe.

If the notes indicate a range, report the mean (e.g. mild-moderate pain as a 1.5).

If otherwise not well documented, annotate as NA.

For situations in which there are multiple sources of truth within this window, select the one closest to week 12.

**Date** **W12AbdPain** (MM/DD/YYYY):

Indicate the date of the note that the daily abdominal pain score was abstracted from. In the event that multiple dates contributed to a score, select the date closest to the date of infusion.

**W12Diarrhea** (Numeric, NA):

Within the week 10-14 period with respect to the patient’s dose of IV Ustekinumab, indicate the total number of bowel movements per day as reported at any point. If the notes report a range, indicate the average of the range. For instance, if the notes indicate 3-4 bowel movements daily, report 3.5 here.

Of note, this definition slightly defers from the trial protocol definition, which indicates the number of liquid or very soft stools daily. The rationale for this difference is that clinical notes tend to better capture total bowel movement frequency than they do characterize the quality of the bowel movement. Many patients with active Crohn’s disease tend to have loose bowel movements. Lastly this distinction is unlikely to impact downstream analyses, especially those that look for within-subject differences over time.

For situations in which there are multiple sources of truth within this window, select the one closest to week 12.

If the notes do not explicitly indicate number of bowel movements during this period, but if notes from before and after this period indicate a stable frequency of bowel movements, then can use heuristics to report this during the baseline period.

If the patient has an ostomy, then annotate this as NA and flag this chart (these may ultimately be excluded). If the patient has an ileal pouch-anal anastomosis (also known as a ‘J-Pouch’, a surgery following complete removal of the rectum and colon), ok to report the number of bowel movements but also flag this chart. If otherwise not well documented, annotate as NA.

**Date** **W12Diarrhea** (MM/DD/YYYY):

Indicate the date of the note that the stool frequency was abstracted from. In the event that multiple dates contributed to a score, select the date closest to the date of infusion.

**W12WellBeing** (Numeric, NA):

Within the week 10-14 period with respect to the patient’s dose of IV Ustekinumab, score the patient’s apparent well-being on the following scale:

0 = generally well, 1 = slightly under par, 2 = poor, 3 = very poor, 4 = terrible

This variable is generally more difficult to annotate and may require some degree of annotator ‘reading between the lines’ in order to perform mapping to this scale. This variable is associated with the existing variables (abdominal pain, diarrhea) but also encompasses other inputs such as fatigue, subjective fevers, anxiety, loss of appetite, and other symptoms.

Patients who are symptom-free should be annotated as a 0. The presence of mild symptoms (occasional abdominal cramping, some loose stools) likely reflects a 1. Patients with significant symptoms, chronic use of opiates, steroids, seeing multiple specialists (mental health, nutrition/dieticians, rheumatology/dermatology) may be annotated as a 2. Patients who are hospitalized should be considered at least a 3, with patients who are suffering concomitant infection/sepsis and/or weight loss considered for a 4. For patients who appear to fit a range (e.g. between a 2 and 3), report the average (e.g. 2.5).

For situations in which there are multiple sources of truth within this window, select the one closest to week 12.

**Date** **W12Wellbeing** (MM/DD/YYYY):

Indicate the date of the note that the wellbeing was abstracted from.

**W12Antidiarrheal** (Y, N, NA): Annotate as Y if the patient has been reported to use either loperamide (i.e. Imodium) or diphenoxylate-atropine (i.e. Lomotil) within the week 10-14 period with respect to the patient’s dose of IV Ustekinumab. If so, annotate as Y. If no, annotate as N. If no explicit comment on the use or absence of antidiarrheals, annotate as NAs. You may find text-searching helpful; consider use of the intentional misspelling ‘immodium’ when searching.

In the event of multiple reports indicating antidiarrheal use, select the one closest to week 12.

Of note, we are anticipating a substantial amount of missing data for this field.

**W12AntidiarrhealDate** (MM/DD/YYYY, NA): Date corresponding to the above.

**W12DailyOpiate** (Y, N, NA): Annotate as Y if the patient had ever used any opiate (oral, subcutaneous, or IV) on a daily basis within the week 10-14 period with respect to the patient’s dose of IV Ustekinumab. These include, but are not limited to: Hydrocodone, Oxycodone, Hydromorphone, Morphine and Codeine. The first two on this list are most common in the outpatient IBD population, including in their formulations that include Acetaminophen (most common brand names Vicodin, Norco).

In the event of multiple reports indicating on opiate use, select the one closest to week 12.

**W12DailyOpiateDate** (MM/DD/YYYY, NA): Date corresponding to the above.

**W12Hct** (Numeric, NA): Within the week 10-14 period with respect to the patient’s dose of IV Ustekinumab, indicate that value here. Use the date that the test was performed, not ordered or resulted. Consider examining scanned clinical documents and Care Everywhere to identify this data, as well as the clinic note where outside lab data is often summarized.

In the event of multiple reports indicating antidiarrheal use, select the one closest to week 12.

If this data is unavailable during this timeframe, indicate NA.

**W12HctDate** (MM/DD/YYYY, NA): Date corresponding to the above.

**W12SteroidUse** (Y, N, NA):

Indicate Y if the patient was taking steroids in the week 10-14 period, N if the patient was not, or NA if insufficient data to determine this.

**W24AbdPain** (Numeric, NA):

Within the week 20-28 period with respect to the patient’s dose of IV Ustekinumab, indicate patient’s level of daily abdominal pain according to the following scale:

0 for none, 1 for mild, 2 for moderate, 3 for severe.

If the notes indicate a range, report the mean (e.g. mild-moderate pain as a 1.5).

If otherwise not well documented, annotate as NA.

For situations in which there are multiple sources of truth within this window, select the one closest to week 24.

**Date** **W24AbdPain** (MM/DD/YYYY):

Indicate the date of the note that the daily abdominal pain score was abstracted from. In the event that multiple dates contributed to a score, select the date closest to the date of infusion.

**W24Diarrhea** (Numeric, NA):

Within the week 20-28 period with respect to the patient’s dose of IV Ustekinumab, indicate the total number of bowel movements per day as reported at any point. If the notes report a range, indicate the average of the range. For instance, if the notes indicate 3-4 bowel movements daily, report 3.5 here.

Of note, this definition slightly defers from the trial protocol definition, which indicates the number of liquid or very soft stools daily. The rationale for this difference is that clinical notes tend to better capture total bowel movement frequency than they do characterize the quality of the bowel movement. Many patients with active Crohn’s disease tend to have loose bowel movements. Lastly this distinction is unlikely to impact downstream analyses, especially those that look for within-subject differences over time.

For situations in which there are multiple sources of truth within this window, select the one closest to week 24.

If the notes do not explicitly indicate number of bowel movements during this period, but if notes from before and after this period indicate a stable frequency of bowel movements, then can use heuristics to report this during the baseline period.

If the patient has an ostomy, then annotate this as NA and flag this chart (these may ultimately be excluded). If the patient has an ileal pouch-anal anastomosis (also known as a ‘J-Pouch’, a surgery following complete removal of the rectum and colon), ok to report the number of bowel movements but also flag this chart. If otherwise not well documented, annotate as NA.

**Date** **W24Diarrhea** (MM/DD/YYYY):

Indicate the date of the note that the stool frequency was abstracted from. In the event that multiple dates contributed to a score, select the date closest to the date of infusion.

**W24WellBeing** (Numeric, NA):

Within the week 20-28 period with respect to the patient’s dose of IV Ustekinumab, score the patient’s apparent well-being on the following scale:

0 = generally well, 1 = slightly under par, 2 = poor, 3 = very poor, 4 = terrible

This variable is generally more difficult to annotate and may require some degree of annotator ‘reading between the lines’ in order to perform mapping to this scale. This variable is associated with the existing variables (abdominal pain, diarrhea) but also encompasses other inputs such as fatigue, subjective fevers, anxiety, loss of appetite, and other symptoms.

Patients who are symptom-free should be annotated as a 0. The presence of mild symptoms (occasional abdominal cramping, some loose stools) likely reflects a 1. Patients with significant symptoms, chronic use of opiates, steroids, seeing multiple specialists (mental health, nutrition/dieticians, rheumatology/dermatology) may be annotated as a 2. Patients who are hospitalized should be considered at least a 3, with patients who are suffering concomitant infection/sepsis and/or weight loss considered for a 4. For patients who appear to fit a range (e.g. between a 2 and 3), report the average (e.g. 2.5).

For situations in which there are multiple sources of truth within this window, select the one closest to week 24.

**Date** **W24Wellbeing** (MM/DD/YYYY):

Indicate the date of the note that the wellbeing was abstracted from.

**W24Antidiarrheal** (Y, N, NA): Annotate as Y if the patient has been reported to use either loperamide (i.e. Imodium) or diphenoxylate-atropine (i.e. Lomotil) within the week 20-28 period with respect to the patient’s dose of IV Ustekinumab. If so, annotate as Y. If no, annotate as N. If no explicit comment on the use or absence of antidiarrheals, annotate as NAs. You may find text-searching helpful; consider use of the intentional misspelling ‘immodium’ when searching.

In the event of multiple reports indicating antidiarrheal use, select the one closest to week 24.

Of note, we are anticipating a substantial amount of missing data for this field.

**W24AntidiarrhealDate** (MM/DD/YYYY, NA): Date corresponding to the above.

**W24DailyOpiate** (Y, N, NA): Annotate as Y if the patient had ever used any opiate (oral, subcutaneous, or IV) on a daily basis within the week 20-28 period with respect to the patient’s dose of IV Ustekinumab. These include, but are not limited to: Hydrocodone, Oxycodone, Hydromorphone, Morphine and Codeine. The first two on this list are most common in the outpatient IBD population, including in their formulations that include Acetaminophen (most common brand names Vicodin, Norco).

In the event of multiple reports indicating on opiate use, select the one closest to week 24.

**W24DailyOpiateDate** (MM/DD/YYYY, NA): Date corresponding to the above.

**W24Hct** (Numeric, NA): Within the week 20-28 period with respect to the patient’s dose of IV Ustekinumab, indicate that value here. Use the date that the test was performed, not ordered or resulted. Consider examining scanned clinical documents and Care Everywhere to identify this data, as well as the clinic note where outside lab data is often summarized.

In the event of multiple reports indicating antidiarrheal use, select the one closest to week 24.

If this data is unavailable during this timeframe, indicate NA.

**W24HctDate** (MM/DD/YYYY, NA): Date corresponding to the above.

**W24SteroidUse** (Y, N, NA):

Indicate Y if the patient was taking steroids in the week 20-28 period, N if the patient was not, or NA if insufficient data to determine this.
